# Supplementary material for: New Copper Complexes with Antibacterial and Cytotoxic Activity
Source: Int J Mol Sci. 2023 Sep 7;24(18):13819. doi: 10.3390/ijms241813819 (PMC10530662; doi:10.3390/ijms241813819)
Supplement: Supplementary file 1 [file ijms-24-13819-s001.zip › ijms-2574527-supplementary.pdf]

Supplementary material

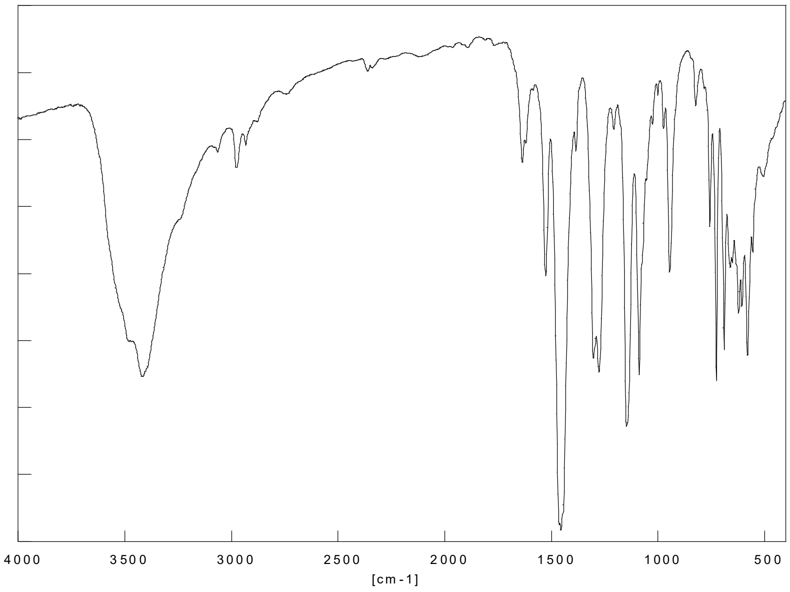

Figure S1. IR spectrum of the C1 complex.

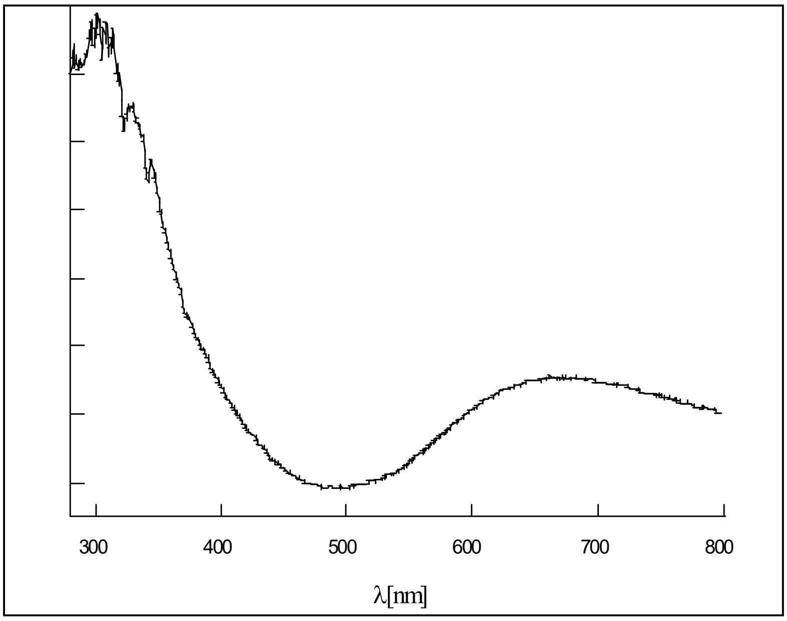

Figure S2. Diffuse UV-Vis reflection spectrum of the C1 complex.

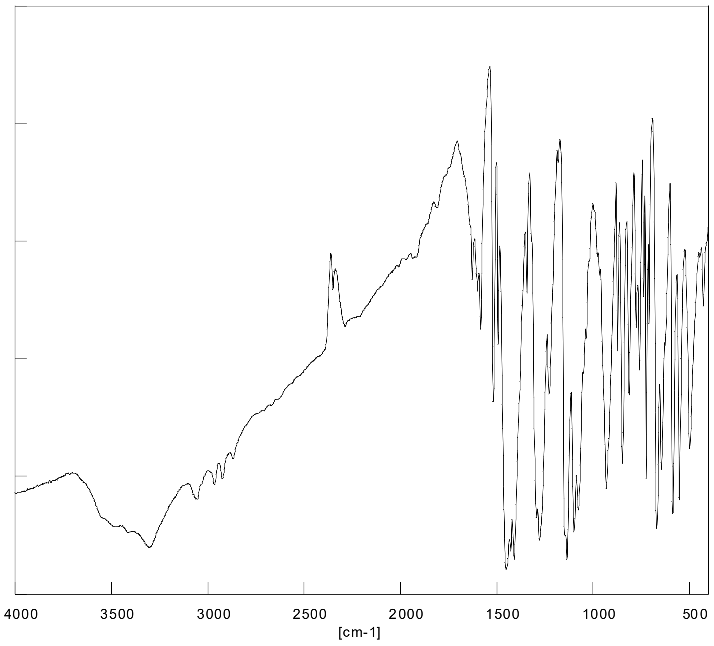

Figure S3. IR spectrum of the C2 complex.

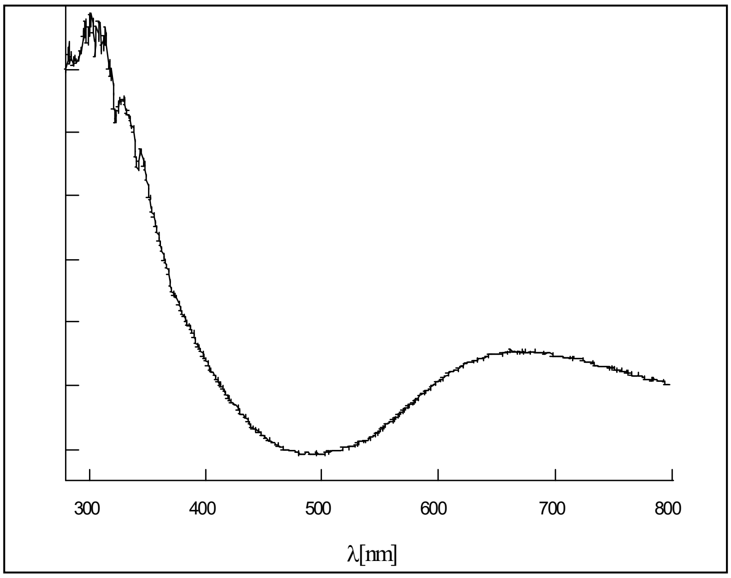

Figure S4. Diffuse UV-Vis reflection spectrum of the C2 complex.
